# Feasibility of a 4 French resuscitative endovascular balloon occlusion of the aorta (REBOA) device for nontraumatic cardiac arrest in a randomized controlled study using a large porcine model

**DOI:** 10.1016/j.resplu.2024.100710

**Published:** 2024-07-15

**Authors:** Adam Power, Asha Parekh, John Landau, Joao Rezende-Neto

**Affiliations:** a Department of Surgery, Western University, London, Ontario, Canada; b School of Biomedical Engineering, Western University, London, Ontario, Canada; c Trauma and Acute Care General Surgery, Department of Surgery, St. Michael’s Hospital, Toronto, Ontario, Canada

**Keywords:** Cardiac arrest, ROSC, CPR, Aortic occlusion

## Abstract

**Aim:**

The objectives of this study were to assess the return of spontaneous circulation rates and hemodynamic response of large swine (>65Kg) during cardiopulmonary resuscitation after nontraumatic cardiac arrest using the COBRA-OS® aortic occlusion balloon and to address limitations of large swine closed-chest cardiopulmonary resuscitation by comparing closed-chest vs. open-chest cardiopulmonary resuscitation.

**Methods:**

Yorkshire pigs (*n* = 10) weighing >65 kg were anesthetized and ventilated. After 7 min of untreated ventricular fibrillation (VF), animals were randomized to receive mechanical closed-chest cardiopulmonary resuscitation or open-chest cardiac massage. Following a 5-minute low-flow state, advanced cardiac life support algorithms were started and the COBRA-OS® was inflated in the thoracic aorta. Animals that achieved return of spontaneous circulation were re-started on mechanical ventilation and medications, CPR, defibrillation, and aortic occlusion were discontinued. The primary outcome was return of spontaneous circulation and secondary outcomes were mean arterial pressures generated in the low flow and aortic occlusion states before return of spontaneous circulation. Groups were compared with a *t*-test or Mann-Whitney *U* test for normal and non-parametric data, respectively, while categorical data was compared with the chi square test.

**Results:**

Return of spontaneous circulation was obtained in 4 animals (80%) in the open cardiac massage group and none in the mechanical closed-chest CPR group (*p* < 0.05). The COBRA-OS® successfully occluded all aortas and animals experienced higher mean arterial pressures in both groups with aortic occlusion (median 15 mm Hg, IQR 13–23 mm Hg), but with a higher MAP difference in the open cardiac massage group (−12.2 mm Hg, [−2.581, −21.819]).

**Conclusions:**

Consideration should be given to intra-thoracic cardiac massage to increase cardiopulmonary resuscitation effectiveness and therefore return of spontaneous circulation rates in large (>65 kg) swine models of nontraumatic cardiac arrest. The COBRA-OS® demonstrated feasibility for use in this model.

The Keenan Research Center, Li Ka Shing Knowledge Institute of St. Michael’s Hospital Animal Care Committee: ACC Protocol #726.

## Introduction

Out-of-hospital cardiac arrest (OHCA) has had a historically poor prognosis, with survival estimated at between 5–10% depending on presenting rhythm and initiation of bystander cardio-pulmonary resuscitation (CPR).^1^, ^2^ Recently, multiple studies have been conducted to evaluate the benefit of ECMO (extracorporeal membrane oxygenation) assisted CPR (E-CPR) to improve survival in OHCA, but results have been heterogeneous.^3^, ^4^, ^5^ ECMO requires a resource and time-intensive therapy, and the systems of care to support a successful and effective E-CPR program may limit it from having generalizability as a tool in cardiac arrest. Resuscitative endovascular balloon occlusion of the aorta (REBOA) has been explored as an alternative adjunct for hemodynamic support in CPR.^6^, ^7^, ^8^, ^9^, ^10^, ^11^, ^12^, ^13^ In comparison to ECMO, REBOA is less resource intensive, can be initiated more efficiently, and has the potential to be more generalizable as an adjunct to CPR.

The COBRA-OS® (Front Line Medical Technologies Inc., London, Ontario, Canada) is the lowest profile aortic occlusion device commercially available (4 French). It is designed to occlude the aorta in the descending thoracic aorta (zone 1; solid black marker; indicating 48  cm depth) or to occlude the aorta below the renal arteries (zone 3; three black markers; indicating 28  cm depth). Its overall working length and therefore maximal reach is 55  cm. The device consists of a stiff stainless-steel inner guidewire with an atraumatic floppy distal J-tip that is housed in a 25 mm compliant occlusion balloon with proximal and distal necks. No other guidewires are required and this is not an over-the-wire device. The COBRA-OS® may be employed in pre-hospital settings due its simplicity and ease of use, potentially allowing less skilled practitioners to use aortic occlusion as an adjunct for OHCA management. Femoral access is known to be the most difficult step in performing REBOA and the 4 French sheath used with this device is approximately the same diameter and placed similarly as most commercially available femoral arterial line monitoring kits.

A clinically relevant animal model of nontraumatic cardiac arrest (NTCA) would be one that adequately mirrors human cardiovascular anatomy in size and shape and would have consistently high rates of return of spontaneous circulation (ROSC) with resuscitative measures used in the clinical setting. Animal studies using swine models of cardiac arrest have consistently demonstrated the physiologic benefits of REBOA as an adjunct to CPR to augment cerebral and coronary perfusion but have had variable rates of (ROSC) (50–100%), with the trend of smaller animals having higher rates of ROSC than larger animals.^14^, ^15^, ^16^, ^17^, ^18^, ^19^, ^20^, ^21^, ^22^, ^23^

The primary objective of this study was to assess the ROSC rates and hemodynamic response of large swine (>65Kg) during CPR after NTCA using the COBRA-OS® as an adjunct to augment proximal blood pressure in an animal model with aortic dimensions comparable to humans.^24^ The secondary objective was to address the limitations of closed-chest CPR in large swine given the angulation and thickness of sternum. Therefore, we compared the ROSC rates and hemodynamic response to closed-chest CPR vs. open-chest CPR in a randomized fashion.

## Methods

### Overview

**Fig. 1:**
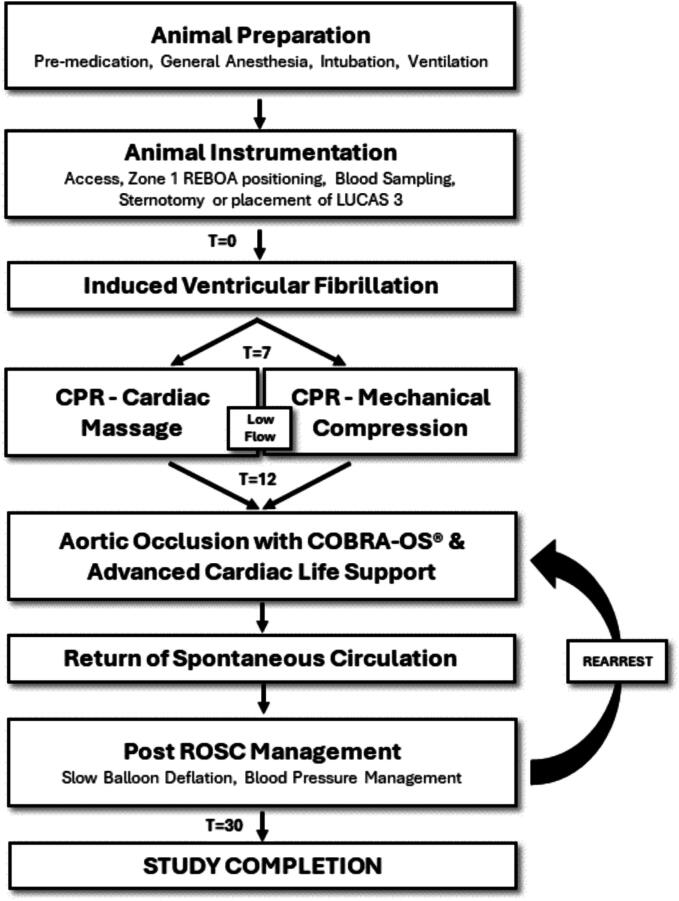
Experimental Procedure.

The Keenan Research Center in the Li Ka Shing Knowledge Institute of St. Michael’s Hospital Animal Care Committee granted approval for this study (ACC Protocol #726) and the National Research Council's Guide for the Care and Use of Laboratory Animals was followed. Ten healthy adult pigs were used in the study (castrated male or nonpregnant females; Yorkshire strain; Source Lifetime Solutions; weight >65 kg). The animals arrived at the animal facility 3–5 days before the planned intervention and were fasted overnight before the procedure. The experimental design, shown in Fig. 1, was modeled after protocols previously published.^15^

### Animal preparation

Ten pigs were pre-medicated with ketamine (20 mg/kg, 6–7 mL), Xylazine (2 mg/kg, 3–3.5 mL), and atropine sulphate (1 mg/25 kg, 1–2 mL), via intramuscular injection using a 21-gauge needle. Following endotracheal intubation, general anesthesia was maintained with 2–5% isoflurane and pigs received 5 mL/kg crystalloids intravenously. The animals were mechanically ventilated with tidal volumes of 10 mL/kg, a positive end-expiratory pressure of 4 cmH_2_O, and a respiratory rate of 10–15 breaths per minute.

The following access sites were obtained via Seldinger technique under ultrasound guidance: 9 French sheath in the right or left internal jugular veins for medications, fluids, and placement of the transvenous pacer (TVP); 7 French sheaths in the left femoral and carotid arteries to monitor proximal and distal mean arterial pressure (MAP); and 4 French sheath in the right femoral artery for REBOA catheter placement (COBRA-OS®). The COBRA-OS® was advanced to zone 1 (between the left subclavian and celiac artery, below the lower heart margin) under fluoroscopy and the balloon was left uninflated. Electrocardiogram monitor and defibrillator pads were placed on all animals. A midline sternotomy was performed in the animals that received open cardiac massage and the pericardial sac was opened. Baseline laboratory and arterial blood gas parameters were obtained from arterial blood sampling from the left femoral arterial line after instrumentation.

Closed-chest CPR was performed either with the LUCAS 3 device (Stryker, Kalamazoo, MI, USA) for the mechanical CPR group set at 100 compressions per minute and compression depth of 5 cm or by two-handed anterior-posterior open cardiac massage at a rate of 100 compressions per minute using a metronome set to 100 per minute. Animals were randomized using a web based random order generator to receive CPR by closed-chest mechanical means or by open-chest cardiac massage prior to intervention.

### Intervention

The TVP was initially placed through the internal jugular sheath into the right ventricle under fluoroscopy and connected to a 9-volt battery. The TVP was pulled back multiple times until electricity was delivered to the AV node which induced ventricular fibrillation (VF) in all animals. Mechanical ventilation and isoflurane were stopped (*T* = 0 min). Following a 7-minute no-flow period, mechanical CPR or open cardiac massage was performed to induce a low-flow state. The ventilator was restarted and set to 10 breaths per minute with 100% oxygen. Following a 5-minute low-flow state, ACLS algorithms were started, and the COBRA-OS® aortic occlusion balloon was inflated to occlusion, which was confirmed with loss of distal femoral arterial pressure waveforms. Defibrillation was performed every 2 min (200 J, biphasic), intravenous 0.01 mg/kg epinephrine was given every 3 min, and ROSC was checked every 2 min as per the Advanced Cardiac Life Support (ACLS) protocol. If ROSC was not obtained after 30 min, all interventions were stopped. In contrast, animals that achieved ROSC were re-started on mechanical ventilation with isoflurane, while the ACLS medications, CPR and defibrillation were discontinued. Aortic damage was assessed macroscopically through gross pathological assessment of the aorta where the balloon was inflated.

If ROSC was achieved, the COBRA-OS® was slowly deflated over 5 min and left in zone 1 of the aorta. Any subsequent cardiac arrest prompted re-initiation of ACLS protocols and COBRA-OS® Zone 1 re-inflation. If ROSC was re-obtained, a strategy of slow deflation of the balloon over 5 min was performed again. If ROSC was not re-obtained by the 30-minute mark, all interventions were stopped. All interventions were stopped at the 30-minute mark in animals that survived the procedures, and the animals were euthanized.

### Data acquisition and analysis

The primary endpoint was return of spontaneous circulation (ROSC) and secondary endpoints were mean arterial pressures (MAPs) generated in the low flow and aortic occlusion pre-ROSC states. The heart rate, blood pressure, ETCO2, respiratory rate, and SpO2 were collected in real-time by using a video camera to record the monitor and data was entered from 30 s intervals subsequently. Arterial blood gases were obtained initially, after ventricular fibrillation arrest, and at study end. An a priori sample size calculation was performed to determine the minimum number of animals for adequate study power using the following parameters: 10% anticipated incidence of ROSC in the mechanical CPR group and 90% in the cardiac massage group with an alpha error 0.05 and 80% power. Groups were compared with a *t*-test or Mann-Whitney *U* test for normal and non-parametric data, respectively, while categorical data was compared with the chi square test. Data was considered statistically significant if *p* < 0.05.

## Results

Baseline characteristics included animal weight, sex, aortic diameters, basic bloodwork (electrolytes and arterial blood gas parameters) and mean arterial pressures. There were no significant differences between baseline data in randomized animals as shown in Table 1. Animals in both groups experienced higher MAPs with aortic occlusion compared to low flow CPR (median 15 mm Hg, IQR 13–23 mm Hg). The COBRA-OS® successfully occluded all aortas with no flow confirmed distally and there were no ruptures of the balloon or any damage to the aorta in the animals and no adverse events.

**Table 1:** Baseline Demographic and laboratory parameters of the mechanical CPR (*n* = 5) and open cardiac massage (*n* = 5) groups.

Parameter	Mechanical CPR (*n* = 5)	SD	Open Cardiac Massage (*n* = 5)	SD	p
Weight (kg)	72.24	3.79	73.64	7.02	0.70
Sex (m:f)	3:2	n/a	2:3	n/a	0.53
Aortic Diameters (mm)	16.2	0.84	16.4	1.34	0.78
Sodium (mmol/L)	141	1.22	140	1.30	0.35
Potassium (mmol/L)	3.52	0.51	3.26	0.05	0.29
Chloride (mmol/L)	107	3.91	106	2.00	0.50
Calcium ion (mmol/L)	0.64	0.18	0.69	0.12	0.68
Glucose (mmol/L)	5.3	0.97	7.1	1.96	0.11
Lactate (mmol/L)	1.56	0.33	1.28	0.55	0.35
pH	7.34	0.03	7.36	0.04	0.59
pCO_2_ (mm Hg)	46	8.02	46	4.14	0.93
pO_2_ (mm Hg)	309	73.00	269	86.94	0.46
SBC (mmol/L)	22.26	2.44	23.96	1.27	0.20
cBase(Ecf) (mmol/L)	741	2.28	744	1.00	0.071
MAP (mm Hg) baseline	66	8.67	64	7.246	0.67

The data following CPR initiation is shown in Table 2, comparing mechanical closed-chest CPR to open cardiac massage. ROSC was obtained in 4 animals (80%) in the open cardiac massage group and none in the mechanical closed-chest CPR group (*p* < 0.05). In the open cardiac massage group, animals had significantly higher MAPs in the low-flow state and aortic occlusion state pre-ROSC compared to the closed-chest mechanical CPR group (mean difference −12.2 mm Hg, [−2.581, −21.819]). Animals in the closed-chest mechanical CPR group had higher final arterial lactate concentrations and a higher amount of epinephrine required than the open cardiac massage group.

**Table 2:** Mechanical CPR versus Open Cardiac Massage ROSC data.

Parameter	Mechanical CPR (*n* = 5)	SD	Open Cardiac Massage (*n* = 5)	SD	p	Mean Difference	95% CI
ROSC (#)	0	n/a	4	n/a	0.0476	n/a	n/a
Rearrest (#)	0	n/a	2 (50%)	n/a	n/a	n/a	n/a
Lactate (mmol/L) no flow	1.72	0.59	1.48	0.64	0.555	0.24	[-0.659, 1.139]
Lactate (mmol/L) final	17.6	5.60	9.22	2.72	0.0167	8.38	[1.967, 14.793]
MAP Pre-ROSC (low flow)	26.2	5.81	48.4	15.77	0.0061	–22.2	[-9.079, −39.321]
MAP Pre-ROSC (aortic occlusion)	39.4	5.86	73.8	24.04	0.0145	−34.4	[-8.888, −59.912]
MAP difference (mm Hg)	13.2	2.59	25.4	11.28	0.009128	−12.2	[-2.581, −21.819]
Total Epinephrine Dose (mcg/kg)	0.1	n/a	0.05	0.032	0.0077	0.05	[0.017, 0.083]

In the 20 min post-ROSC, 2/4 (50%) animals in the open cardiac massage group re-arrested with VF after a period of declining MAP and required re-inflation of the COBRA-OS® in Zone 1. Both animals achieved ROSC again and stayed alive until the end of the study. All 4 animals with ROSC had sinus rhythm at study conclusion.

## Discussion

REBOA is a promising technique being studied to potentially improve ROSC rates for OHCA, with a human randomized controlled trial currently enrolling patients in Europe.^25^ Our findings showed that the COBRA-OS® improved the hemodynamic response in REBOA-assisted CPR in a large animal model resulting in higher proximal blood pressure and ROSC rates. Moreover, there was no evidence of balloon rupture, aortic injury, or failure to successfully achieve aortic occlusion. Due to the 4 French diameter of the COBRA-OS® (negating the need to upsize an initial sheath) and advancement method without the use of an over-the-wire technique, endovascular aortic occlusion can be obtained promptly, as quickly as 70 s in a previous study.^26^

As aortic occlusion techniques continue to be refined, devices will require testing to ensure their safety and effectiveness. It is therefore imperative to investigate those features in clinically relevant animal models that adequately reflect human anatomy to allow for more direct translation of results. Furthermore, in keeping with the reduction principle in the 4R Rule in laboratory animal science, it is also important to keep animal wastage to a minimum. In previous cardiac arrest porcine models, using larger animals often came at the expense of requiring more animals due to decreased ROSC rates (See Table 3). From our previous work, we have shown the average aortic diameter is 15 mm in large porcine models (>65 kg), which is comparable to the average human aortic diameter that is approximately 20mm.^27^ This study is the first to demonstrate acceptable ROSC rates (80%) in a large (>65 kg) swine model with REBOA-assisted non-traumatic cardiac arrest and the use of open cardiac massage to mitigate the anatomic limitations of closed-chest CPR in swine.

**Table 3:** Comparison of ROSC rates in varying swine sizes.

Year	Authors	Species	# of Animals	Mean weight (kg)	ROSC Rate (%)
2023	Nakashima et al	Yorkshire mix swine	24	52	83
2023	Tiba et al	Yorkshire mix swine	8	50.4	87.5
			8	52	75
			8	51.8	100
2022	Nowadly et al	Sus scrofa	10	72	50
			10	71	50
2021	Olsen et al	Sus scrofa domesticus	10	49	90
2020	Nowadly et al	Sus scrofa	5	70	100
			5	70	100
			5	62.5	100
2020	Dogan et al	Hampshire and Yorkshire	35	32	91
			30	32	98
2019	Tiba et al	Yorkshire mix	6	43	50
2019	Dogan et al	Hampshire and Yorkshire	18	28	17

Our findings showed that closed-chest mechanical CPR had a diminished effect on MAP and ROSC during ACLS compared to open cardiac massage, which may be due to the anatomy of the sternum and chest wall of large swine. Interestingly, this also correlates with previous human studies that have demonstrated that the use of mechanical CPR in cardiac arrest is not associated with improved ROSC rates.^28^ Our results indicate that future studies intended to evaluate ROSC during CPR using large swine should consider intrathoracic cardiac massage. This approach may allow for higher rates of ROSC, thereby decreasing the number of animals used in the study.

This study highlights the need for improved options to treat real world out-of-hospital cardiac arrest. Mechanical CPR has been deemed a good option for this patient population since its emergence, especially for emergency medical service professionals, and use of these devices significantly grew with its availability; one study revealing a 4-fold increase in the use of mechanical CPR devices among patients with out-of-hospital cardiac arrest treated by emergency medical services professionals between 2010–2016 in the United States.^29^ These devices may have benefits for end-user ease of use, but come at a significant cost, and subsequent data has shown that there is still a gap to improving patient outcomes.^28^

In our study, 50% of the animals experienced rearrest, which was reversed by re-inflating the balloon. This reversal could be partially explained by improvement in cerebral and coronary perfusion pressures with the insufflation of the balloon in the aorta.^31^ Early human data suggests that balloon deflation after a period of elevated afterload from REBOA may increase the risk of re‐arrest.^30^ The optimal time to perform balloon deflation is currently unknown and post-ROSC strategies such as extended deflation times, intermittent zone 1 balloon inflation, and automated or fixed partial zone 1 balloon inflation would be valuable areas of further study in this regard.

Our study has several limitations. First, the use of intrathoracic cardiac massage limits the overall generalizability of the results to humans since it is not used in ACLS protocols. Second, previous studies have demonstrated that improving the diastolic blood pressure specifically may be a more important variable than MAP in the generation of ROSC and therefore should have been reported. Third, these animals did not have pre-existing cardiac disease, which is usually present in humans that suffer from NTCA, which may limit direct translation. Finally, the LUCAS 3 device may not be the best mechanical compression device for these large animals due to its intended use for human chests, although most other studies investigating REBOA and NTCA have employed its use. Despite these limitations, this is the first study to propose an alternative strategy for improving ROSC rates in a large animal NTCA REBOA model.

## Conclusion

Consideration should be given to intra-thoracic cardiac massage to increase CPR effectiveness and therefore ROSC rates if a large swine model (>65 kg) is used to study NTCA based on our findings. The COBRA-OS® demonstrated feasibility for use in this model.

## Funding

This work was supported in part by the National Research Council of Canada Industrial Research Assistance Program (Project number 998620). They had no involvement in study design; in the collection, analysis and interpretation of data; in the writing of the report; or in the decision to submit the article for publication.

## CRediT authorship contribution statement

**Adam Power:** Writing – review & editing, Writing – original draft, Visualization, Methodology, Investigation, Formal analysis, Data curation, Conceptualization. **Asha Parekh:** Writing – review & editing, Project administration, Methodology, Investigation, Funding acquisition, Conceptualization. **John Landau:** Writing – review & editing, Investigation. **Joao Rezende-Neto:** Writing – review & editing, Resources, Investigation.

## Declaration of competing interest

The authors declare the following financial interests/personal relationships which may be considered as potential competing interests: [Adam Power and Asha Parekh are co-founders and have an equity stake in Front Line Medical Technologies Inc. John Landau received consulting fees related to this study. Joao Rezende-Neto is a co-founder and has an equity stake in InventorrMD; InventorrMD received consulting fees related to this study.].
